# GPR55 and PINK1 as emerging therapeutic targets in Glioma: insights into their modulation by phytochemicals and plant extracts

**DOI:** 10.3389/fphar.2026.1760169

**Published:** 2026-07-24

**Authors:** Andres David Turizo Smith, Jean Carlos Martinez Perez, Juan Camilo Marin Loaiza, Gonzalo Arboleda

**Affiliations:** 1 Departamento de Patología, Facultad de Medicina, Universidad Nacional de Colombia, Bogotá, Colombia; 2 Grupo de investigación INFARMA-HUS, Hospital Universitario de la Samaritana, Bogotá, Colombia; 3 Grupo de Investigación en Fitoquímica y Farmacognosia de la Universidad Nacional de Colombia, Departamento de Farmacia, Facultad de Ciencias, Universidad Nacional de Colombia, Bogotá, Colombia; 4 Departamento de Farmacia, Facultad de Ciencias, Universidad Nacional de Colombia, Bogotá, Colombia

**Keywords:** *Cannabis sativa*, glioma, GPR55, PINK1, Piper nigrum, plant extract

## Abstract

The expression of certain genes and proteins in brain tumors, particularly gliomas, presents potential therapeutic targets and biomarkers. One of such genes encodes the protein PINK1 (PTEN-induced Kinase 1), which has been primarily studied due to its mutations being linked to Parkinson’s disease (PD). Additionally, epidemiological studies have observed a reduced risk of developing certain types of cancer in patients with neurodegenerative diseases and vice versa. The GPR55 receptor (G protein-coupled receptor 55) exhibits pro-oncogenic properties, and its expression correlates with tumor aggressiveness and the activation of the MAPK/ERK signaling pathway. This review would aim to provide an overview of the current understanding of the relationship between PINK1 and GPR55 proteins in glioma pathophysiology and explore the potential use of plant extracts as a therapeutic alternative to regulate their expression.

## Introduction

Gliomas are the most prevalent tumors in the CNS. They are a heterogeneous group of tumors with distinctive histopathological characteristics that generally present as a diffuse and aggressive intracerebral mass. Gliomas originate from glial progenitor cells, which develop and grow, resembling astrocytic and/or oligodendroglial lineages (Delgado-López et al., 2020; Zong et al., 2012). As with many types of cancer, there are no clearly defined etiological factors, except associated to certain genetic mutations that predispose individuals to glioma formation; and following ionizing radiation used in radiotherapy treatments (Delgado-López et al., 2020).

From a histopathological perspective, gliomas can be classified into two domains. First, low-grade glioma (LGG), which tends to have a good prognosis after surgical resection and adjuvant radiation and/or chemotherapy (Delgado-López et al., 2020; Delgado-López et al., 2017) and second, high-grade glioma (HGG), which also benefits from maximal surgical resection and mandatory postoperative chemoradiation. However, while therapy for LGG currently can extend life expectancy to 10–15 years on average from diagnosis (Delgado-López et al., 2020; Duffau, 2018), aggressive treatment for the more common HGG, glioblastoma multiforme (GBM), results in a modest overall survival of 14 months. The reasons for this poor prognosis in HGG include its infiltrative nature, limiting disease-free survival even in cases of supramaximal resection, and increased resistance to current therapies mainly related to lack of complete knowledge about biochemical and signaling pathways that drive their physiopathology (Delgado-López et al., 2020). Therefore, it is imperative to find new therapeutic approaches to improve prognosis and survival for this type of cancer.

The pathways regulated by the PTEN-induced kinase 1 (PINK1) and the G protein-coupled receptor 55 (GPR55) represent such new potential therapeutic targets in gliomas. It has been reported that PINK1 inhibits GBM growth by regulating mitochondrial oxidative phosphorylation, aerobic glycolysis, and reactive oxygen species (ROS) (O’Flanagan and O’Neill, 2014); while GPR55 has been observed to promote proliferation, and its expression correlates with tumor aggressiveness (Ross, 2011).

This review aims to critically examine the current evidence regarding the interplay between PINK1 and GPR55 in glioma pathophysiology, and to evaluate the therapeutic potential of plant-derived compounds in modulating their expression. Given the emerging and heterogeneous nature of the available data, a structured narrative review methodology was adopted. The search strategy, inclusion criteria, and methodological limitations are described in the following section.

### Search strategy and selection criteria

This narrative review follows the SANRA guidelines to ensure transparency. A comprehensive search was conducted (2014–2024) in PubMed, ScienceDirect, Web of Science, and Scopus.1.1. Search StrategyWe employed a combination of MeSH terms and free-text keywords focused on four pillars: PINK1 in Glioma: (e.g., “PINK1”, “PARK6”, “PTEN-induced putative kinase 1”). GPR55 in Glioma: (e.g., “GPR55”, “G protein-coupled receptor 55”). Phytochemicals: (e.g., “cannabinoids”, “piperine”, “natural products”). Contextual: (e.g., “Colombia”, “Latin America”, “glioma” OR “natural products”). Search strings were adapted to each database. While no initial language limits were set, final selection focused on English-language full-texts.

### Selection and synthesis

From an initial pool of 1,247 records, two independent reviewers (ADTS, JCM) screened titles and abstracts. Discrepancies were resolved by a third reviewer (GA). Finally, 82 articles were selected for synthesis, comprising: 1. Original Research (n = 45): Mechanistic data on PINK1/GPR55. 2. Reviews (n = 22): Theoretical framework. 3. Clinical/Epidemiological (n = 5): Trials (e.g., NCT03529448) and GLOBOCAN data. 4. Snowballing (n = 25): Additional seminal works identified from bibliographies.

### Critical appraisal and data extraction

Data extraction focused on molecular targets (PI3K/AKT/mTOR, mitochondrial dynamics), glioma models, and phytochemical efficacy. Given the preclinical nature of most evidence, studies were appraised based on translational relevance (clinically relevant concentrations and orthotopic models) and methodological rigor.

## Integration of authors’ preliminary data

Preliminary *in silico* and *in vitro* data from our research group (Turizo-Smith et al., unpublished observations) are referenced in the context of cannabinoid modulation of PINK1 and GPR55. These data are explicitly identified as unpublished preliminary findings and are interpreted cautiously in light of established literature. They are included to illustrate emerging hypotheses rather than as conclusive evidence, in accordance with narrative review conventions.

## PINK1, GPR55, and plant extracts in gliomas

### PINK1

The PTEN-induced putative kinase 1 (PINK1) gene contains eight exons and encodes a protein of 581 amino acids, which consists of a non-canonical N-terminal mitochondrial targeting sequence (MTS), a transmembrane domain, a highly conserved serine/threonine kinase domain, and a C-terminal autoregulatory sequence (O’Flanagan and O’Neill, 2014). PINK1 shows homology with the Ca2+/calmodulin kinase family, most closely related to CaMKI, CaMKII, and the myotonic dystrophy protein kinase (DMPK) (O’Flanagan and O’Neill, 2014). PINK1 is ubiquitously expressed and localized in mitochondria and cytosol, with higher levels reported in the CNS, heart, testis, liver, and skeletal muscle (O’Flanagan and O’Neill, 2014).

PINK1 has been shown to have important pro-survival, anti-apoptotic, and cytoprotective functions, with PINK1 exerting primary activities on mitochondrial homeostasis, including the control of mitophagy, bioenergetics, fission, and fusion (O’Flanagan and O’Neill, 2014). On the other hand, current evidence indicates that PINK1 acts as a tumor promoter and mediator of chemotherapy resistance, having a dual role in cancer biology with both pro- and anti-tumor properties depending on the context (O’Flanagan and O’Neill, 2014; Wang et al., 2022).

In addition, PINK1 interacts with the pivotal oncogenic signaling axis mediated by the PI3K/AKT/mTOR signaling pathway, mediating critical mitochondrial and metabolic functions that regulate cancer cell survival, growth, stress resistance, and the cell cycle. Through its activation of AKT, PINK1 has a cytoprotective and chemotherapy-resistant function, as it regulates mitochondrial homeostasis, in particular by enhancing anaerobic glycolysis (O’Flanagan and O’Neill, 2014; Wang et al., 2022).

FOXO is a family of transcription factors whose activation generally results in cell cycle arrest or cell death. One of its members, FOXO3a, controls the transcription of PINK1 in response to growth factor deprivation (O’Flanagan and O’Neill, 2014; Hagenbuchner and Ausserlechner, 2013). In addition, PINK1 suppresses ROS generation and tumor growth through FOXO3a, which acts as a master regulator of oxidative stress through regulation of superoxide dismutase 2 (SOD2), thereby attenuating GBM growth (O’Flanagan and O’Neill, 2014; Hagenbuchner and Ausserlechner, 2013; Luo et al., 2018b).

**FIGURE 1:**
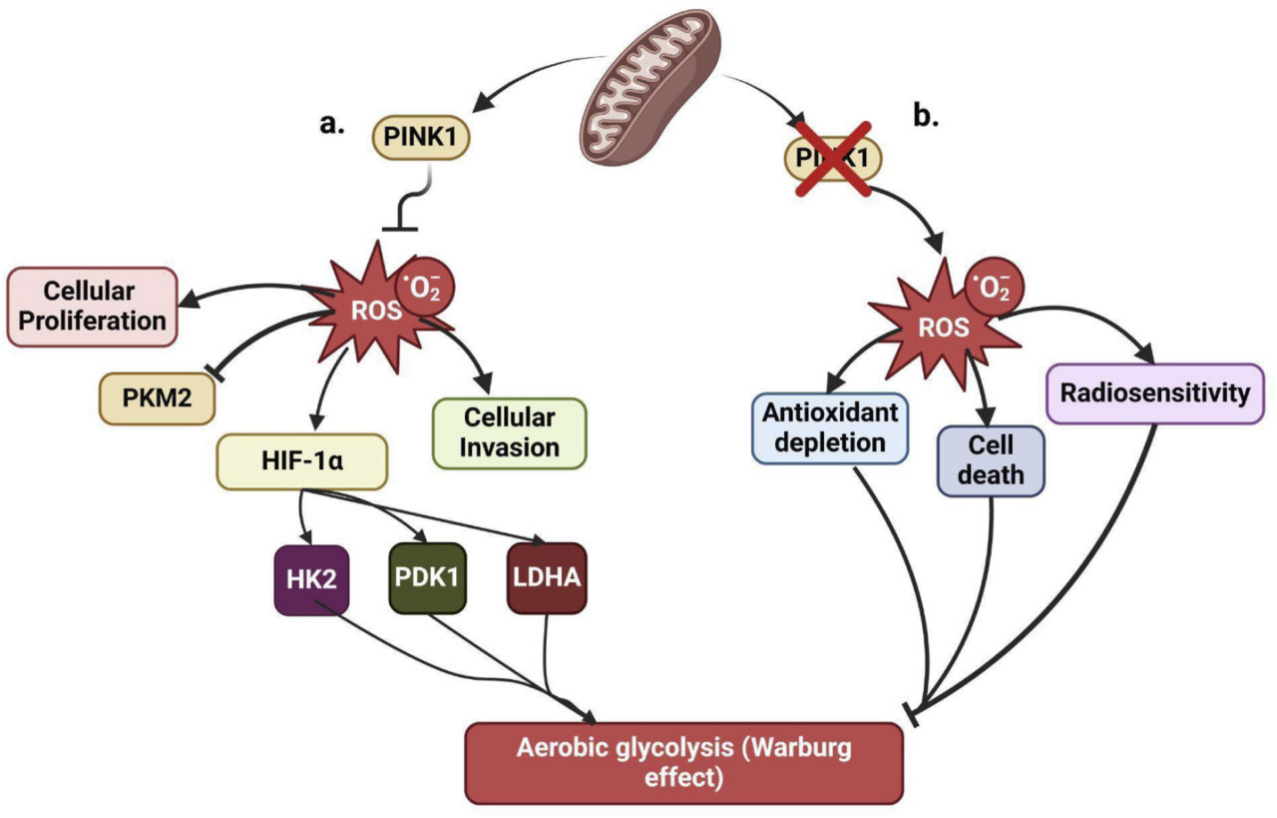
Summary of PINK1 Function in Gliomas. **(a)** PINK1 in GBM Cells Suppresses Reactive Oxygen Species (ROS). In GBM cells, PINK1 plays a crucial role in the regulation of ROS generation. When PINK1 function is intact, it prevents the accumulation of ROS, which would inhibit pyruvate kinase M2 (PKM2), a key enzyme in glycolysis. Reduced PKM2 activity supports tumor cell proliferation, partly by enhancing the pentose phosphate pathway (PPP). Additionally, this process stabilizes hypoxia-inducible factor alpha 1 (HIF-1α), leading to modulation of hexokinase 2 (HK2), pyruvate dehydrogenase kinase 1 (PDK1), and lactate dehydrogenase A (LDHA). This modulation initiates glycolytic metabolism, known as the Warburg effect, favoring glycolysis over oxidative phosphorylation to sustain rapid tumor growth and cellular invasiveness. Therefore, proper PINK1 function helps control and deplete carcinogenic growth. **(b)** Loss or Downregulation of PINK1 in GBM Cells Increases ROS. The downregulation of PINK1 in GBM cells results in elevated ROS levels, triggering oxidative stress. This oxidative stress induces cell death and depletes antioxidants, such as NADH and glutathione (GSH), which in turn sensitizes the cells to radiation therapy. Biorender was used to construct the figure.

The loss of PINK1 elicits variable responses in different cell types and contexts. It has been demonstrated that loss of PINK1 expression in neurons and various other cell types induces cell death (Hagenbuchner and Ausserlechner, 2013; Luo et al., 2018b). However, loss of PINK1 has been observed in a significant number of human brain tumors, including GBM, which correlated with poor patient survival. When PINK1 expression is reduced, it causes the stabilization of HIF-1α (hypoxia-inducible factor alpha) through elevated ROS levels, which leads cancer cells to increase aerobic glycolysis and produce higher levels of oxidative stress via ROS (Figure 1). These cells, in turn, strictly regulate the generation, accumulation, and elimination of ROS to avoid cell death (Luo et al., 2018b; Agnihotri et al., 2016; Ferber et al., 2011).

Overexpression of PINK1 attenuated *in vivo* GBM growth in mouse orthotopic xenografts, by regulating mitochondrial oxidative phosphorylation, aerobic glycolysis, and ROS generation. These findings are consistent with previous studies demonstrating that lower levels of ROS scavenging enzymes, including SIRT3, may promote aerobic glycolysis and metabolic reprogramming in both normal and cancer cells, while its overexpression reduces cancer cell growth (Agnihotri et al., 2016). In addition, the impact of PINK1 on mitophagy may be associated with desensitization of glioma cells to conventional therapies such as surgery, chemotherapy, and radiotherapy, worsening the patient’s prognosis (Qu et al., 2019).

The functional role of PINK1 in cancer biology presents a therapeutic paradox that demands careful contextual interpretation (O’Flanagan and O’Neill, 2014). Initially characterized as a tumor suppressor through its regulation of mitochondrial quality control, emerging evidence reveals a conditional oncogenic potential dependent on cellular context, metabolic state, and PTEN status (Wang et al., 2022).

In glioblastoma, PINK1 predominantly exerts antitumorigenic effects through three interconnected mechanisms: (i) FOXO3a-mediated metabolic reprogramming, where PINK1 enhances mitochondrial oxidative phosphorylation and suppresses aerobic glycolysis (Warburg effect) (Agnihotri et al., 2016); (ii) ROS homeostasis, via stabilization of SOD2 and attenuation of HIF-1α accumulation under normoxic conditions (Agnihotri et al., 2016); and (iii) mitophagy-dependent quality control, preventing accumulation of damaged mitochondria that would otherwise generate genotoxic ROS (Delgado-López et al., 2020). Agnihotri et al. demonstrated that PINK1 loss correlates with significantly poorer survival in GBM patients, with PINK1-deficient tumors exhibiting enhanced glycolytic metabolism and aggressive growth in orthotopic xenografts (Agnihotri et al., 2016). Overexpression studies confirm this suppressive role: PINK1 restoration in GBM cells attenuates tumor growth *in vivo* by normalizing mitochondrial bioenergetics (Agnihotri et al., 2016).

Conversely, PINK1 activation of the PI3K/AKT/mTOR survival axis can promote chemotherapy resistance and cell survival under metabolic stress (O’Flanagan and O’Neill, 2014; Wang et al., 2022). This apparent contradiction resolves when considering context-dependent determinants: (i) Baseline mitochondrial stress: In cells with pre-existing mitochondrial dysfunction, PINK1-mediated mitophagy may selectively eliminate damaged organelles, paradoxically enhancing survival of stressed cancer cells (Qu et al., 2019); (ii) PTEN mutational status: In PTEN-deficient gliomas, constitutive PI3K/AKT activation may override PINK1’s metabolic tumor suppression, unmasking survival-promoting functions (Wang et al., 2022); (iii) Hypoxic microenvironment: HIF-1α stabilization in hypoxic regions may shift PINK1 signaling toward cytoprotective autophagy rather than tumor suppression (Ferber et al., 2011); and (iv) Therapeutic intervention: PINK1-dependent mitophagy induced by radiation or chemotherapy may protect glioma cells from treatment-induced apoptosis, contributing to therapeutic resistance (Qu et al., 2019).

The available evidence suggests that PINK1 functions could be working predominantly as a tumor suppressor in GBM (Agnihotri et al., 2016), but this function becomes compromised or inverted under specific pathological conditions. The determinants of functional outcome include the balance between mitochondrial biogenesis and clearance, the redox state of the tumor microenvironment, and oncogenic driver mutations. For therapeutic targeting, this duality implies that PINK1 restoration (gene therapy, mitophagy enhancers) may benefit patients with PINK1-deficient, glycolytic tumors, whereas PINK1 inhibition may sensitize resistant tumors to conventional therapy (Wang et al., 2022; Qu et al., 2019).

### GPR55

GPR55 is a member of the G protein-coupled receptor (GPCR) family, specifically the purinergic subfamily (Ye et al., 2019). GPR55 plays significant roles in signal transduction from the external environment to the intracellular milieu. It is expressed in diverse regions of the CNS including the caudate nucleus, putamen, hippocampus, thalamus, frontal cortex, and hypothalamus. It is also expressed in the adrenal glands, dorsal root ganglion, endothelial cells, and gastrointestinal tract. GPR55 binds to Ga12/13 and Gq proteins. Several studies have highlighted the role of Ga12/13 signaling in promoting cancer cell proliferation, invasion, and metastatic dissemination (Ross, 2011). Additionally, elevated GPR55 mRNA expression has been associated with the aggressiveness of certain tumor types such as GBM in humans (Singh et al., 2016). It was first demonstrated that L-α-lysophosphatidylinositol (LPI) is an agonist of GPR55 (Ross, 2011), inducing rapid phosphorylation of extracellular signal-regulated kinase (ERK) and an increase in intracellular Ca2+ levels (Ross, 2011).

GPR55 is expressed in many human cancer cell line models, and together with LPI, it has been associated with proliferation, angiogenesis, migration, and invasion of breast, ovarian, prostate, and GBM cancer cells *in vitro*. In contrast, downregulation of the GPR55 produces the opposite effect (Alhouayek et al., 2018). Studies also indicate that GPR55 has an oncogenic role in cancer progression *in vivo*. For instance, the elimination of GPR55 reduces tumor growth in xenografted mice; and GPR55-deficient mice were more resistant to carcinogen-induced carcinomas and azoxymethane-induced colorectal cancer (Alhouayek et al., 2018; Hasenoehrl et al., 2017). In another study, mice injected with MDA-MB-231 breast cancer cells, which endogenously express GPR55, showed more cases of lung metastasis when treated with LPI compared to the vehicle, an effect that was modulated when GPR55 was knocked out in the injected cells (Alhouayek et al., 2018).

In other studies, inhibition of GPR55 function with (R,R′)-4′-methoxy-1-naphthylphenol (MNF) or with an antagonist like CID16020046 results in a decrease in the levels of multidrug resistance (MDR) proteins such as P-glycoprotein (Pgp) in total cell extracts, as well as a reduction in nuclear expression of Pgp and breast cancer resistance protein (BCP). Additionally, in these models, the concomitant use of chemotherapeutic agents like doxorubicin in PANC-1 cells increases cytotoxicity (Singh et al., 2016).

**FIGURE 2:**
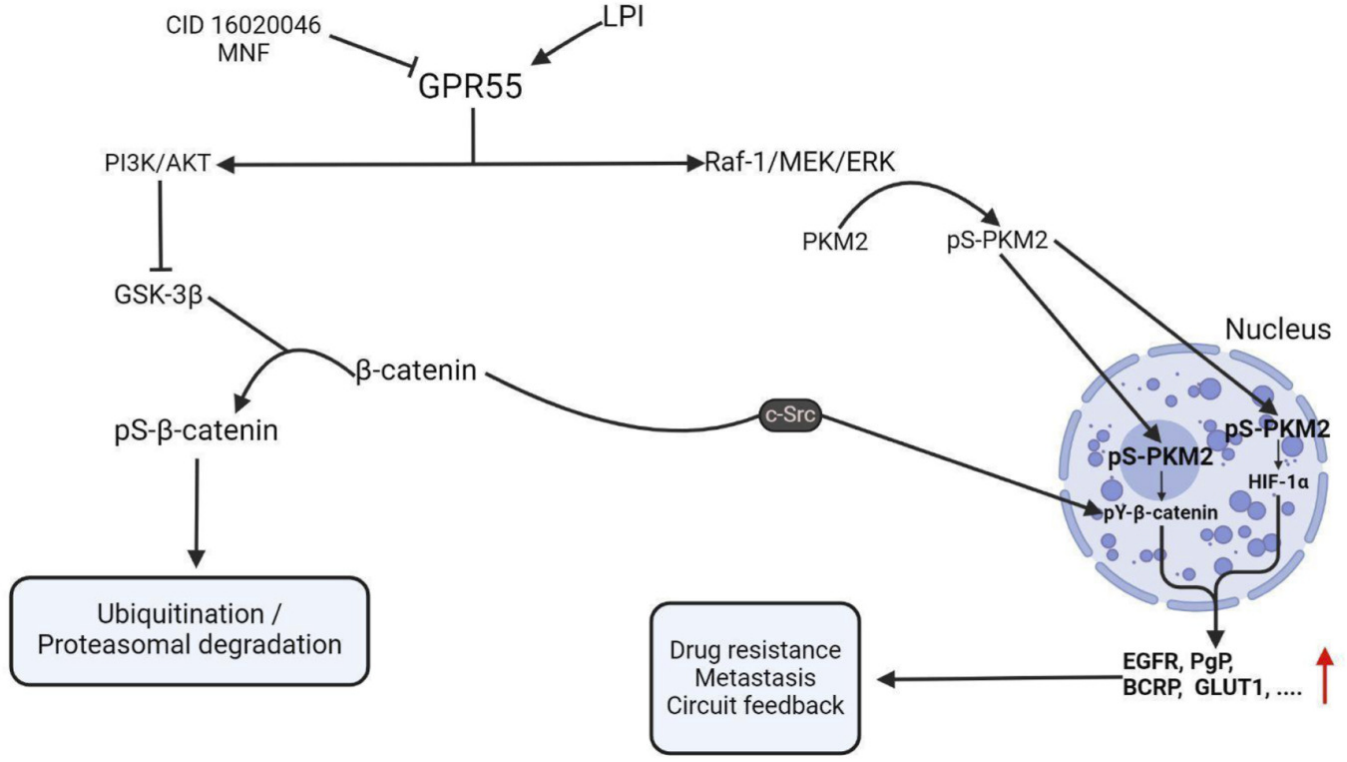
Mechanistic Pathway of GPR55-Mediated Signaling in Tumor Progression and Drug Resistance Modulation. The schematic illustration depicts the signaling pathway activated by lysophosphatidylinositol (LPI) through G protein-coupled receptor GPR55, which initiates downstream signaling associated with tumor progression. Upon activation, GPR55 triggers the PI3K/AKT pathway, a critical axis for cell survival and proliferation, which inhibits GSK-3β (glycogen synthase kinase 3 beta). This inhibition stabilizes β-catenin, a transcriptional co-activator, preventing its degradation through ubiquitination and proteasomal degradation. Stabilized β-catenin can then translocate to the nucleus and regulate gene expression related to cell survival, metastasis, and drug resistance. The pathway also involves the enzyme pyruvate kinase M2 (PKM2), which, upon phosphorylation (pS-PKM2), moves to the nucleus where it interacts with hypoxia-inducible factor 1-alpha (HIF-1α), a transcription factor that supports adaptation to hypoxia and upregulation of drug resistance genes. The interaction between phosphorylated PKM2 and β-catenin is mediated by the tyrosine kinase c-Src, further enhancing the transcriptional activation of tumor progression-related genes. This signaling cascade ultimately leads to the expression of genes such as EGFR, Pgp, BCRP, and GLUT1, which contribute to drug resistance mechanisms (e.g., Pgp and BCRP as efflux transporters) and increased glucose uptake (GLUT1). This pathway highlights a potential mechanism through which GPR55 antagonists may exert antitumor effects by interfering with tumor cell survival and resistance. Biorender was used to construct the figure.

In U87MG GBM cells, which express high levels of GPR55, data suggest that inhibiting GPR55 activity produces antitumor effects by attenuating the MEK/ERK and PI3K/AKT pathways, leading to a reduction in the expression and function of MDR proteins. In these models, GPR55 antagonism attenuates both the constitutive and ligand-induced activity of these pathways, resulting in reduced phosphorylation and nuclear translocation of pyruvate kinase muscle isozyme 2 (PKM2) and a concomitant decrease in HIF-1α and β-catenin levels. This significantly reduces MDR expression levels, leading to decreased chemoresistance and tumor survival (Figure 2) (Singh et al., 2016).

Although most of these studies indicate that the activation of GPR55 may have a procarcinogenic role, it has been observed that anandamide and O-1602 exert antiproliferative effects in cholangiocarcinoma in a GPR55-dependent manner (Alhouayek et al., 2018). Therefore, more studies are needed to more precisely define the role of GPR55 in carcinogenic processes.

### Functional selectivity and ligand-specific signaling

GPR55 exhibits ligand-dependent functional diversity wherein different ligands activate distinct signaling pathways with opposing biological outcomes (Ross, 2011; Ye et al., 2019; Alhouayek et al., 2018). This property complicates therapeutic targeting, as the net effect depends on the specific ligand-receptor interaction rather than mere receptor activation.

The endogenous lipid L-α-lysophosphatidylinositol (LPI) activates Gα12/13-RhoA-ERK signaling, promoting proliferation, migration, and invasion (Ross, 2011; Alhouayek et al., 2018). In U87MG GBM cells, LPI induces ERK phosphorylation and increases intracellular Ca^2+^ (Ross, 2011). GPR55-deficient mice show reduced tumor growth and resistance to carcinogen-induced carcinomas (Alhouayek et al., 2018; Hasenoehrl et al., 2017), supporting an oncogenic role for LPI-GPR55 signaling.

Conversely, anandamide and the atypical cannabinoid O-1602 exert antiproliferative effects in cholangiocarcinoma via GPR55-dependent mechanisms (Alhouayek et al., 2018). Similarly, cannabigerol (CBG) functions as a GPR55 antagonist in glioblastoma stem cells, inducing cell cycle arrest and apoptosis (Lah et al., 2021; Lah et al., 2022). Importantly, receptor crosstalk modulates GPR55 oncogenicity: concurrent activation of the β2-adrenergic receptor (β2-AR) combined with GPR55 blockage synergistically disrupts pro-oncogenic signaling in glioma cells, suggesting that the net mitogenic effect of GPR55 depends on the broader receptor network context (Wnorow et al., 2017). This illustrates that GPR55 signaling is not isolated but integrated with adrenergic and potentially other GPCR systems in glioma pathophysiology.

The divergent effects likely reflect: (i) G protein coupling preferences, with Gα12/13 favoring proliferation and alternative couplings (Gαq, Gα13) mediating distinct responses (Ross, 2011); (ii) receptor modulation by heterodimerization with other cannabinoid receptors (Ye et al., 2019); and (iii) competitive antagonism by phytocannabinoids at the LPI binding site (Lah et al., 2022; Wnorow et al., 2017). The expression of interacting partners (CB1, CB2, β2-AR) in specific glioma subtypes may further determine signaling outcomes (Ye et al., 2019; Wnorow et al., 2017).

This ligand-specificity and receptor crosstalk necessitate differentiated therapeutic strategies: antagonists (CID16020046, MNF, CBG) for LPI-driven tumors (Singh et al., 2016; Lah et al., 2021; Lah et al., 2022), combinatorial approaches targeting GPR55-β2-AR axes (Wnorow et al., 2017), and potentially GPR55-targeted cytotoxic ligands for resistant phenotypes. Patient stratification based on tumor lipid profile, adrenergic receptor status, and cannabinoid receptor expression may optimize therapeutic targeting.

### Plant extracts and cancer

Despite significant biological and clinical advancements and the associated costs, it is crucial to identify new treatment strategies to improve outcomes for patients with GBM (Weller et al., 2017; Silva et al., 2019). Various bioactive products derived from plants have been reported to prevent the tumorigenesis of different types of tumors (Silva et al., 2019; Cragg and Pezzuto, 2015). Therefore, in the search for new therapies, research on plant extracts and their antineoplastic mechanisms has emerged as a promising and successful field (Silva et al., 2019). Numerous alternative plant-based treatments and therapies have been explored, which is particularly important for patients who cannot tolerate the side effects of conventional therapy.

Although the research on the application of plant extracts as therapeutic agents against cancer is relatively new, approximately half of the conventional chemotherapy agents have plant origins, with about 25% directly derived from plants and another 25% being chemically modified versions of plant derivatives (Amin et al., 2009; Sugier et al., 2019). This fact has driven extensive efforts to identify anticancer properties in plants and chemically characterize them to reveal the presence of many bioactive compounds, such as polyphenols, taxols, and brassinosteroids (Sugier et al., 2019). Several types of malignancies, including glioma models, have been reported to decrease following treatment with plant extracts (Sugier et al., 2019; Lesgards et al., 2014) (Table 1).

**TABLE 1:** Summary of studies on plant extracts, fractions, and derived compounds in various glioma cell lines and potential effects/mechanisms of action involved.

Agent or chemical compound used	Plant species (Bibliographic Reference)	Cell lines	Effects
α-Bisabolol	Matricaria chamomilla, (Cavalieri et al., 2004)	T67, U87	Apoptosis inducer (intrinsic pathway)
IngC, Ingenol-3-angelate	Euphorbia tirucalli, (Alhouayek et al., 2018)	U87MG, GMAG, SF188	Protein kinase inhibitor
2,5-Dimethoxy-p-cymene	Arnica montana (Sugier et al., 2019)	T98G, MOGGCCM	Pro-apoptotic
β-Elemene	Curcuma wenyujin, (Zhao et al., 2011)	C6 glioma cells, U87MG	Hsp90/Raf-1
Oleuropein	Olea europaea, (Tezcan et al., 2019)	T98G	mRNA modulation
Crata BL	Crataeva tapia, (Bonturi et al., 2019)	U87/MSC	Cell proliferation inhibitor/pro-apoptotic
Whole Extract	Curcuma raktakanda, (Mishra et al., 2019)	C6 glioma cells	Bcl-xL, inhibitory effects on NF-κB activation
Whole Extract	Calystegia sepium, (Rezadoost et al., 2019)	U87MG	Pro-apoptotic
Curcumin	Curcuma longa, (Heebkaew et al., 2019)	U373MG, U87MG	Autophagy (LC3-II agonism)
18-Nor-ent-labdan	Grazielia gaudichaudeana, (Balbinot et al., 2021)	U251	Cytotoxic, unspecified**
Whole Extract	*Centella asiatica*, (Viswanathan et al., 2019)	C6 glioma cells	MAPK pathway modulator
Ginsenosides	Panax ginseng, (Ham et al., 2019)	528NS	Inhibition of Wnt/β-catenin signaling
Whole Extract	Phyllanthus taxodiifolius, (Kwanthongdee et al., 2019)	C6 glioma cells	Inhibitor of adhesion, migration, and invasion
Isolinderalactone	Lindera aggregata, (Hwang et al., 2018)	U87	BCL-2/XIAP inhibitor (activates caspase-3/DNA damage), tumor growth suppressor via enhanced apoptosis
Whole Extract (polyphenolic and terpenoid compounds)	Menyanthes trifoliata, (Kowalczyk et al., 2019)	GBM cells	Apoptosis inducer (modulates Bax, Bcl-2, Cas-3, p53, and decreases mitochondrial potential)
Whole Extract (carotenoids, crocins) trans-crocin 1 (56.4%), trans-crocin 2 (19.2%), cis-crocin 1 (6.8%)	Crocus sativus, (Giakoumettis et al., 2018)	C6 glioma cells	Autophagy (nuclear p53 modulation)
Saponin-rich fraction	Agave sisalana, (De Oliveira et al., 2018)	C6 glioma cells	Cytotoxicity through cytoplasmic vacuole formation
Forskolin	Plectranthus barbatus, (Lv et al., 2018)	U87, A172	Pro-apoptotic
Cannabidiol Δ9-Tetrahydrocannabinol	*Cannabis sat*iva, (Kis et al., 2019)	U373, U87/GSC3832, GSC387	Pro-apoptotic (TPRV2, 5HT1a)
Cannabigerol (CBG)	*Cannabis sativa*, (Lah et al., 2021)	U87MG, U373MG, T98G, glioblastoma stem cells (GSC)	Reduces cell viability, induces apoptosis, and inhibits invasion. Shows similar effectiveness to CBD and temozolomide in inhibiting glioma spheroid invasion. Particularly effective in combination with CBD for inhibiting the viability of glioma and GSC cells
Cannabigerol (CBG) Cannabidiol (CBD)	*Cannabis sativa*, (Lah et al., 2022)	U87MG, U373MG, T98G, GSC	CBG acts as an antagonist to the GPR55 receptor, which is highly expressed in GSCs and involved in tumorigenic signaling through pathways such as MAPK/ERK. Inhibition of GPR55 by CBG disrupts this pathway, leading to cell cycle arrest in the G1 phase and triggering intrinsic apoptosis via caspase-3/7 activation. CBG also desensitizes the TRPV1 receptor, reducing calcium influx and promoting autophagy and
​	​	​	apoptosis.CBD binds to allosteric sites on the CB1 receptor and modulates TRPV1 and GPR55 activity. Through CB1, CBD induces endoplasmic reticulum stress and activates eIFKA3, leading to autophagy via mTOR inhibition. CBD’s antagonistic action on GPR55 prevents the activation of downstream MAPK/ERK signaling and cell proliferation. This results in cell cycle arrest and caspase-dependent apoptosis, specifically targeting glioma stem cell markers such as SOX2 and CD15, ultimately suppressing GSC stemness and tumorigenicity
Δ9-Tetrahydrocannabinol (THC)	*Cannabis sativa.* (Kolbe et al., 2023)	Patient-derived glioblastoma cells	THC acts via the GPR55 receptor, activating PLC-IP3 signaling pathways and, in some cases, RhoA-ROCK signaling in a cell-type-specific manner. Activation of the PLC-IP3 pathway generates IP3, which mobilizes intracellular Ca^2+^ from the endoplasmic reticulum, while the RhoA-ROCK pathway contributes to cytoskeletal regulation. In GBM #10 cells, both PLC and RhoA-ROCK pathways work together to reduce proliferation. Inhibition of these pathways using U73122 (for PLC) and Y-27632 (for ROCK) blocks THC’s effects on cell proliferation, suggesting a combined PLC- and ROCK-dependent mechanism in GPR55-mediated signaling
α, β momorcharin	Momordica charantia, (Manoharan et al., 2013)	U87MG, 1321N1	Pro-apoptotic via cytochrome c release, increased caspase 3 and 9
Eufol	Euphorbia tirucalli, (Salehi et al., 2019; Galvez et al., 1993)	GAMG	Inhibition of proliferation and migration
Hesperetin	Origanum majorana, (Erenler et al., 2015; Gutiérrez-Grijalva et al., 2017)	C6 glioma cells	Antiproliferative
Benzyl isothiocyanate (derived from benzyl glucosinolate)	Tropaeolum majus, (Guzmán-Pérez et al., 2016; Zhu et al., 2013)	U87MG	Pro-apoptotic/antiproliferative
Anthocyanin-rich extract	Euterpe oleracea, (Hogan et al., 2009; Alessandra-Perini et al., 2018)	C6 glioma cells	Pro-apoptotic (Downregulation of Bcl-2 and upregulation of caspase 3)
Gibbilimbol B Eriopodol Erioquinol	Piper eriopodon, (Muñoz et al., 2019)	U373	Pro-apoptotic (XIAP antagonist via BIRD3 inhibition, caspase 9, 3/7, and ROS increase inducing caspase-independent cell death)
Sinomenine SW33	Sinomenium acutum, (Zheng et al., 2021)	U87MG, U251	Mitochondrial inhibition, leading to cytochrome C and caspase release, as well as G2/M phase arrest in the cell cycle. Additionally, the PI3K/AKT/mTOR and AMPK/mTOR signaling pathways are involved, activating autophagy
7α-acetoxy-6β-hydroxyroyleanone (Roy)	Plectranthus hadiensis, (Domínguez-Martín et al., 2022)	U87, A172, U118, U373, H4	Induces DNA double-strand breaks, G2/M cell cycle arrest, apoptosis, decreased mitochondrial membrane potential, and modulates the expression of pro-apoptotic and anti-apoptotic genes (BAX, BCL2, TP53, and CAS3)
Ganostile supplement (Miconet s.r.l.) in combination with Cisplatin and Pt (IV)Ac-POA	Ganoderma lucidum Eleuterococcus senticosus Echinacea purpurea Astragalus embranaceus, Gaiaschi et al. (2024)	U251 cell line and fibroblast	Antiproliferative and antitumoral actions related to proteins such as PINK1
Curcumin	Curcuma longa L Lin et al. (2024)	GBM cells	Inhibited PI3K/Akt/mTOR pathway to activate autophagy and inhibit mitophagy and related proteins such as PINK1

Among the studies examining the effects of plant extracts or their components on glioma cells, those showing the most promising effects include:

1. α-bisabolol: this natural, non-toxic compound induces strong apoptosis in glioma cells. It has shown potential as an effective treatment in preclinical studies (Sugier et al., 2019; Cavalieri et al., 2004).
2. Thymol (timol): it has been demonstrated to have an inhibitory effect on cell growth in the human GBM cell line DBTRG-05MG. It is known for its antimicrobial and antioxidant properties (Sugier et al., 2019; Lesgards et al., 2014; Cavalieri et al., 2004; Hsu et al., 2011).
3. β-elemene: it is a natural compound extracted from Curcuma wenyujin. It has shown promising anticancer effects against a wide spectrum of tumors, including inducing apoptosis in glioblastoma cells (Sugier et al., 2019; Mu et al., 2016; Zhao et al., 2011).
4. Euphorbia tirucalli: a plant widely used in Brazilian traditional medicine against cancer, has shown cytotoxic activity in various types of human cancer cell cultures. One specific compound derived from this plant, ingenol-3-dodecanoate (IngC), has demonstrated potential as an antitumoral agent against gliomas. IngC appears to be a major modulator of various isoforms of protein kinase C, promoting cell cycle arrest in the S phase and inducing autophagy in gliomas (Silva et al., 2019). In other studies involving this plant, eufol, a tetracyclic triterpenoid compound, has shown particular activity against glioma cells, including pediatric and adult glioma cell lines, with IC50 values ranging from 5.98 to 31.05 µM. This activity is much more potent than temozolomide, a clinical drug used to treat some brain cancers, which has IC50 values ranging from 97.00 µM to 1 mM. Furthermore, eufol exhibits a higher selective cytotoxicity index (0.64–3.36) compared to temozolomide (0.11–1.13) (Salehi et al., 2019).

In the case of plants like *Cannabis sativa,* one of its terpenophenol compounds, cannabidiol (CBD), has demonstrated concentration-dependent inhibition of human glioma cell viability in U87 cells after 24 h of incubation with CBD at 5–10 µM. Additionally, these compounds have shown inhibition of growth in human glioma cell lines U373 and U87 implanted in female athymic nude CD-1 mice (31). Recently, CBD has been shown to inhibit the viability of glioma stem cell (GSC) lines 3832 and 387, inducing apoptosis through ROS production. Furthermore, *in vivo* treatment of intracranial GSC-derived tumors with CBD (15 mg/kg) appears to inhibit tumor cell proliferation through the proapoptotic caspase-3 pathway, resulting in a significant extension of mouse survival. Although GSCs have shown adaptation to CBD treatment, this effect has been suppressed when CBD is combined with small molecule ROS modulators (such as vitamin E). Together, these data suggest that a combination of CBD with vitamin E may regulate ROS levels, representing a promising therapeutic model for managing GBM (Singer et al., 2015).

Additionally, CBD at 0.4 µM has been shown to inhibit the growth of different GBM cell lines (U87-MG, U251, and SF126), suggesting that CBD may be a more potent inhibitor of GBM cell growth than Δ9-tetrahydrocannabinol (THC) (Marcu et al., 2010). In this regard, the role of transient receptor potential (TRP) channels is in regulating cell proliferation and differentiation in these models, demonstrating that CBD appears to act as a specific ligand for TRPV2, by functioning as a selective activator of TRPV2 by enhancing Ca2+ entry in U87MG cells with an IC50 of 22.2 μM. Thus, CBD could be used as a promising therapeutic agent against GBM cancer cell lines (Nabissi et al., 2012).

Other studies have shown that microparticles loaded with cannabinoids (terpenophenols) increase apoptosis and decrease cell proliferation in U87MG glioma cells. Microparticles containing only CBD (6.7 mg) were administered *in vivo* in experimental athymic nude mice (Kis et al., 2019). Alharris et al. demonstrated that CBD at 10 µM induces apoptosis in neuroblastoma cell lines SH SY5Y and IMR-32 through activation of serotonin and vanilloid receptors, significantly reducing cell migration and invasion *in vitro* (Kis et al., 2019; Alharris et al., 2019). In addition, Ryberg et al. reported that the GPR55 receptor can be modulated by a series of ligands derived from *Cannabis sativa* in transfected human embryonic kidney (HEK293s) cells (Ryberg et al., 2007).

**FIGURE 3:**
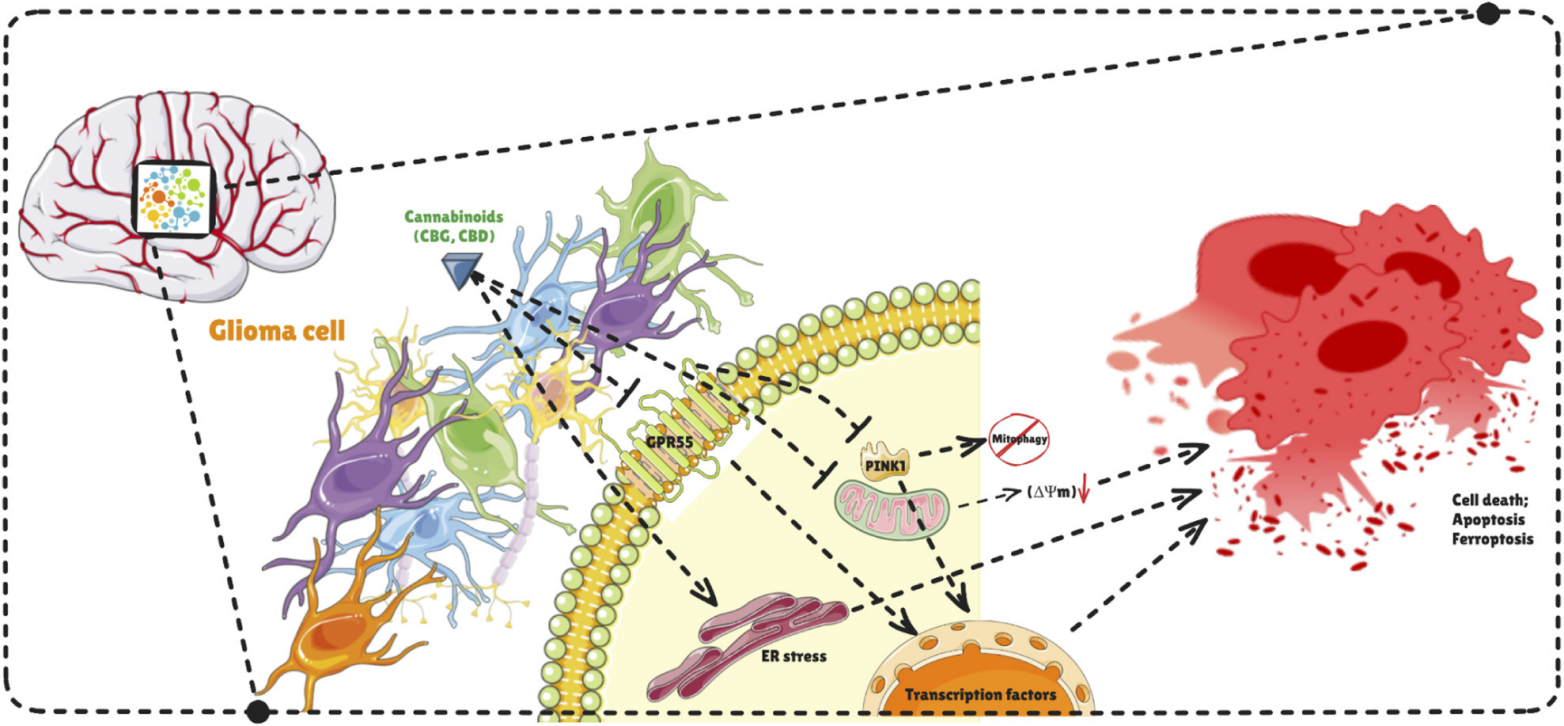
Interplay between PINK1, GPR55, and cannabinoids in the glioma context. This schematic illustrates how modulation of PINK1 and GPR55 by cannabinoids may influence glioma cell survival. Activation or inhibition of these pathways could lead to impaired mitophagy, increased apoptotic signaling, and a reduction in resistance mechanisms typically observed in glioma cells. The figure highlights the potential of targeting mitochondrial quality control via the PINK1 pathway and GPR55 to induce glioma cell death.

It is worthy to mention that our group found that some cannabinoid compounds, such as CBD and CBG, may modulate PINK1 and GPR55 *in silico* models. Furthermore, these compounds could induce PINK1-related cell death by antagonizing its function, leading to a loss of mitochondrial potential and a subsequent decrease in cell viability in astrocytoma and glioblastoma models *in vitro*. Additionally, our research revealed that PINK1 and GPR55 are differentially expressed in glioblastoma patients compared to normal brain tissue, as well as in other cancers. Notably, PINK1 appears to be absent in normal glial cells when compared to glioblastoma, according to our immunohistochemistry (IHC) data. Data mining analyses conducted using public databases such as UALCAN, GEPIA, and the Human Genome Atlas further highlight PINK1 levels as a factor influencing patient survival, depending on the tumor context. This effect is not limited to glioma, but also extends to other cancer subtypes, including pancreatic cancer (PAAD), lung cancer, renal cancer among others (Turizo Smith et al., 2025). These findings are visually summarized in Figure 3, which illustrates the interplay between PINK1, GPR55, and cannabinoids in the glioma context, highlighting the potential of modulating these pathways to impair mitophagy, promote apoptosis, and reduce resistance mechanisms in glioma cells.

Part of the illustration was created using Servier Medical Art (provided by Servier, licensed under a Creative Commons Attribution 3.0 Unported License), and is additionally covered under the Creative Commons Attribution-NoDerivatives 4.0 International License (CC BY-ND 4.0). The apoptosis icon was created by Helicase11 and is licensed under CC BY 4.0.

The plants of the Piper genus (Piperaceae family), traditionally a common food resource in neotropical forests and widely used for culinary spices, also constitute a class of medicinal plants containing phenolic bioactive compounds of interest (Xiang et al., 2016; Muñoz et al., 2019). Among them, piplartine, gibbilimbols A–D, eriopodol, and erioquinol have shown potent cytotoxic/antitumor effects in a variety of human cancer cells both *in vitro and in vivo* (Munoz et al., 2018; Petit et al., 2005). Gibbilimbol B and eriopodol A appear to induce a proapoptotic response through mitochondrial outer membrane depolarization, followed by cytochrome C release and subsequent activation of initiator and effector caspases in U373 cells (Muñoz et al., 2019). This process is mediated by modulation of X-linked inhibitor of apoptosis protein (XIAP). On the other hand, erioquinol seems to provoke non-apoptotic cell death via caspase-independent pathway through cytoplasmic ROS, which may be of interest for simultaneously targeting tumors and improving inflammatory response (Muñoz et al., 2019). XIAP in this context represents an interesting crossroad of pathways involved in both cell death and inflammation.

Finally, regarding the potential regulation of PINK1, these compounds from the Piper genus might regulate PINK1 downstream, as other studies have observed that stable expression of PINK1 in SH-SY5Y cells reduces cytochrome c translocation (Petit et al., 2005). On the other hand, PINK1 deficiency in renal tubular epithelial cells in mice with induced acute kidney injury results in increased caspase 3 and subsequent apoptosis (Lin et al., 2019). These findings may provide clues to the possible correlation between XIAP modulation and PINK1 modulation, which needs to be evaluated and further expanded in the context of gliomas.

An approach taken by our research group was to investigate the effects of *Piper nigrum* extracts and fractions on gliomas and neuroblastomas, considering the specific tumor subtype. While the literature reports antitumor activity of *Piper nigrum* in gastrointestinal and breast cancer models (Liu et al., 2010; Turrini et al., 2020), our results indicate that most fractions exhibit cytotoxic effects in glioma and neuroblastoma cell lines (Turizo Smith et al., 2025). However, some fractions appear to increase cell viability and may even counteract the cytotoxic effects of other compounds (possible efflux pump inhibition (Kumar et al., 2018; Holland et al., 2007)). Notably, purified piperine did not exhibit this cytoprotective effect in tumor cell lines, unlike the *Piper nigrum* extract (Turizo Smith et al., 2025). Additionally, a review of the literature suggests that in various murine models, *Piper nigrum* may exert procarcinogenic effects in the liver, skin, and lungs (Shwaireb et al., 1990; Wrba et al., 1992). These findings raise questions regarding its potential therapeutic benefits and translational application, emphasizing the need for further investigation in more complex models.

### Convergence of PINK1 and GPR55 signaling: a working hypothesis

While PINK1 and GPR55 operate in distinct cellular compartments, mitochondria and plasma membrane, respectively, emerging evidence suggests potential functional crosstalk that remains largely hypothetical in glioma. Here we present a theoretical framework based on pathway convergence rather than established molecular interaction, explicitly distinguishing validated mechanisms from proposed connections.

While both proteins independently regulate core cancer hallmarks in glioblastoma. PINK1 suppresses tumor growth via mitochondrial oxidative phosphorylation, ROS homeostasis, and antagonism of the Warburg effect (Agnihotri et al., 2016). GPR55 promotes proliferation, chemoresistance, and invasive signaling through PI3K/AKT and MAPK/ERK pathways (Ross, 2011). These parallel but separate functions suggest complementary rather than redundant roles in glioma biology.

Three nodes of potential functional interaction warrant investigation:

- Metabolic Reprogramming and the Warburg Effect. Both pathways regulate glycolytic enzyme expression and glucose metabolism. PINK1 suppresses HIF-1α stabilization and downstream glycolytic targets (HK2, PDK1, LDHA) through FOXO3a-mediated ROS control (O’Flanagan and O’Neill, 2014). GPR55 activation, conversely, stabilizes HIF-1α via PI3K/AKT-dependent mechanisms and nuclear PKM2 translocation (Singh et al., 2016). The opposing effects on HIF-1α—PINK1 suppression versus GPR55 promotion suggest a potential metabolic toggle where relative pathway activity determines glycolytic versus oxidative metabolic states.
- Reactive Oxygen Species Homeostasis. PINK1 maintains mitochondrial ROS at non-genotoxic levels through SOD2 regulation and mitophagy (O’Flanagan and O’Neill, 2014). GPR55 signaling influences intracellular calcium and potentially mitochondrial Ca^2+^ uptake (Ross, 2011), which modulates ROS generation at the electron transport chain. However, direct evidence linking GPR55 activation to PINK1-mediated ROS detoxification is absent; this represents a theoretical connection based on shared downstream consequences rather than demonstrated molecular interaction.
- Chemoresistance and Cell Survival. Both proteins modulate sensitivity to conventional glioma therapies. PINK1-dependent mitophagy protects cells from apoptosis induced by radiation and chemotherapy (Qu et al., 2019), while GPR55 upregulates multidrug resistance proteins (P-gp, BCRP) via PI3K/AKT/β-catenin signaling (Singh et al., 2016). The concurrent targeting of both pathways pharmacological PINK1 inhibition to prevent cytoprotective mitophagy, and GPR55 antagonism to reduce efflux pump expression represents a rational but experimentally untested combination strategy.

Critically, no study has demonstrated physical interaction between GPR55 and PINK1, nor has genetic or pharmacological epistasis been established in glioma models. PINK1 lacks canonical GPCR interaction domains. Proposed crosstalk occurs at the level of shared downstream effectors (HIF-1α, metabolic enzymes, survival kinases) rather than direct signaling complex formation.

Future research to uncover the proposed interplay would require experimental validation. In this context, priority research questions include: (a) Does GPR55 activation modulate PINK1 expression, stability, or mitochondrial localization? (b) Does PINK1-dependent mitochondrial quality control affect GPR55 surface expression or signaling bias? (c) Do combined modulations of both pathways produce synergistic, additive, or antagonistic effects in glioma models? (d) How do tumor-specific mutations (PTEN, IDH, EGFR) alter the balance between PINK1 tumor suppression and GPR55 oncogenicity?

Finally, plant-derived compounds targeting both pathways independently such as cannabinoids with dual GPR55 antagonist/PINK1 modulatory properties (Turizo Smith et al., 2025) may offer therapeutic advantages through convergent mechanism disruption. However, claims of “dual targeting” should be interpreted cautiously given the absence of demonstrated pathway interaction; observed effects likely reflect parallel rather than synergistic pathway inhibition.

## Limitations and translational challenges

Despite promising preclinical findings, the translation of PINK1- and GPR55-targeted therapies from bench to bedside faces substantial barriers that must be critically acknowledged (O’Flanagan and O’Neill, 2014; Wang et al., 2022). The predominantly descriptive nature of current phytochemical research in glioma, combined with inherent biological complexities of the central nervous system, necessitates cautious interpretation of available evidence (Delgado-López et al., 2020; Weller et al., 2017).

### Preclinical bias and model limitations

The current evidence base relies overwhelmingly on *in vitro* studies using established glioma cell lines (U87MG, U251, T98G, U373) (Delgado-López et al., 2020; Agnihotri et al., 2016) and subcutaneous xenografts in immunodeficient mice (Weller et al., 2017). These models present critical limitations: (i) lack of tumor heterogeneity established cell lines poorly recapitulate the molecular diversity of patient-derived GBM, particularly the proneural, classical, and mesenchymal subtypes; (ii) absence of immune context immunodeficient models preclude evaluation of immunomodulatory effects, increasingly recognized as central to glioma progression and therapy response (Weller et al., 2017); (iii) non-physiological microenvironment subcutaneous xenografts lack the hypoxic, acidic, and immunosuppressive brain microenvironment that critically influences drug penetration and efficacy (Delgado-López et al., 2020; Weller et al., 2017).

Orthotopic models, though more representative, introduce additional complexity: the murine blood-tumor barrier differs substantially from human GBM vasculature, and species-specific differences in drug metabolism complicate pharmacokinetic extrapolation (Weller et al., 2017). Patient-derived xenografts (PDX) and genetically engineered mouse models (GEMM) offer improved fidelity but remain underutilized in phytochemical studies (Delgado-López et al., 2020). The absence of standardized preclinical criteria such as the NCI’s “Minimum Information About a Cancer Model” guidelines limits cross-study comparability and reproducibility.

### Blood-brain barrier penetration

The blood-brain barrier (BBB) represents the principal obstacle for glioma drug delivery (Wang et al., 2022; Agnihotri et al., 2016; Weller et al., 2017). Formed by tight junctions between brain microvascular endothelial cells, pericytes, and astrocytic endfeet, the BBB excludes approximately 98% of small molecules and nearly all biologics (Wang et al., 2022). Critical challenges specific to phytochemicals include:

Many promising compounds exhibit poor BBB penetration due to: (i) high molecular weight (>400–500 Da); (ii) polar surface area (>60–70 Å^2^); (iii) high hydrogen bond donor/acceptor counts; and (iv) efflux by P-glycoprotein (P-gp) and breast cancer resistance protein (BCRP), highly expressed at the BBB and further upregulated in glioma endothelium (Wang et al., 2022; Singh et al., 2016). For example, curcumin shows negligible brain bioavailability (<1%) after oral administration due to rapid glucuronidation and efflux transport (Lin et al., 2024).

Another situation is that Rodent BBB differs from humans in transporter expression (higher P-gp in mice), tight junction protein composition, and paracellular permeability, complicating extrapolation of brain penetration data (Wang et al., 2022).

The blood-tumor barrier (BTB) in GBM is heterogeneous regions of barrier disruption (“leaky” vasculature) coexist with intact BBB, particularly in infiltrative tumor margins where recurrence originates (Delgado-López et al., 2020; Weller et al., 2017). This spatial heterogeneity means that even compounds achieving therapeutic concentrations in the tumor core may fail to target invasive cells (Weller et al., 2017).

### Pharmacokinetic and bioavailability limitations

Phytochemicals generally exhibit suboptimal pharmacokinetic profiles for CNS drug development (Massi et al., 2003; Kis et al., 2019):

- Low oral bioavailability. Cannabidiol (CBD) demonstrates ∼6% oral bioavailability due to extensive first-pass hepatic metabolism by CYP3A4 and CYP2C19; Δ9-THC shows similarly poor absorption with high interindividual variability. Piperine, despite its bioenhancing properties, undergoes rapid glucuronidation with terminal half-life <1 h in humans (Liu et al., 2010; Kumar et al., 2018).
- High plasma protein binding. Many lipophilic phytochemicals (cannabinoids, terpenes) are >95% bound to plasma proteins, reducing the free fraction available for BBB penetration (Kis et al., 2019).
- Limited metabolic stability. Polyphenols (curcumin, resveratrol) undergo rapid Phase II metabolism; alkaloids (piperine, piplartine) are susceptible to oxidative degradation (Silva et al., 2019; Xiang et al., 2016).
- Dose-exposure nonlinearity. Saturable absorption and hepatic metabolism result in nonlinear pharmacokinetics, complicating dose optimization (Massi et al., 2003).

### Pharmaceutical strategies to overcome delivery barriers

To overcome these pharmacokinetic limitations and achieve effective CNS distribution, advanced pharmaceutical formulations are essential to optimize bioavailability, permeability, and therapeutic efficacy while minimizing systemic toxicity. Liposomal encapsulation represents a primary strategy, significantly improving the solubility and stability of hydrophobic phytochemicals such as CBD. For instance, CBD-loaded liposomes using DSPC/DSPE-PEG formulations achieve encapsulation efficiencies up to 87% and sustained release over 504 h, overcoming the poor oral bioavailability (∼6%) of free CBD (Jurgelane et al., 2025). Cationic liposomal formulations further enhance BBB penetration through electrostatic interactions with endothelial membranes, enabling co-delivery of synergistic combinations such as acteoside with CBD or naringenin, which demonstrate potent apoptotic effects against glioblastoma cells (IC50 ∼5–6 µM) (S et al., 2025). Similarly, liposomal co-delivery of CBD with celecoxib effectively suppresses Wnt/β-catenin and NF-κB signaling pathways while activating Nrf2, inducing apoptosis and oxidative stress in GBM cells (Rybarczyk et al., 2025). Beyond liposomes, peptide-based nanocarriers offer alternative solutions; self-assembling peptide micelles can increase CBD aqueous solubility by 2000-fold through π-π stacking interactions, achieving 80.4% encapsulation efficiency (Parlak, 2024). Additionally, dual receptor-targeting co-drug-loaded nanoparticles specifically designed to penetrate the BBB and inhibit oxidative phosphorylation represent a promising approach for targeted glioblastoma therapy, enabling simultaneous modulation of multiple metabolic pathways (Sun et al., 2023). These nanoformulation strategies collectively address the challenges of BBB penetration, metabolic instability, and poor solubility, transforming promising phytochemicals into viable clinical candidates for glioma therapy.

### Standardization and reproducibility of plant extracts

Chemical heterogeneity represents a fundamental challenge for translational development (Silva et al., 2019; Xiang et al., 2016). Plant extracts exhibit batch-to-batch variability due to: (i) geographic and seasonal variation in secondary metabolite production; (ii) cultivation conditions (soil composition, altitude, light exposure); (iii) harvest time and plant part (roots vs. leaves vs. fruits); and (iv) extraction methodology (solvent polarity, temperature, duration) (Silva et al., 2019; Xiang et al., 2016; Muñoz et al., 2019).

For example, *Cannabis sativa* extracts vary 10–100 fold in cannabinoid content depending on chemotype (THC-dominant, CBD-dominant, balanced); even standardized preparations show significant lot-to-lot variation in terpene profiles that may influence pharmacological activity (Massi et al., 2003; Ryberg et al., 2007). Similarly, *Piper nigrum* alkaloid content varies 3-5 fold between geographic origins (Xiang et al., 2016; Liu et al., 2010).

This variability complicates: (i) reproducibility of preclinical findings across laboratories; (ii) regulatory approval, which requires consistent manufacturing and quality control; and (iii) clinical trial design, where undefined extracts preclude dose-response characterization. The transition from crude extracts to standardized fractions or isolated compounds (e.g., purified CBD vs. Cannabis extract) represents a necessary but economically challenging evolution (Silva et al., 2019; Massi et al., 2003).

### Concentration-response and therapeutic index concerns

Many reported *in vitro* effects occur at concentrations pharmacologically unattainable in human brain tissue (Massi et al., 2003; Kis et al., 2019):

- Discrepancy between *in vitro* and *in vivo* concentrations. CBD cytotoxicity in U87MG cells is reported at 5–50 μM, but free brain concentrations in rodents after systemic administration of 10–30 mg/kg reach only 0.1–1 µM. Similar disconnections exist for thymol (25 µM *in vitro*), β-elemene (40–100 µM), and piperine (50–100 µM) (Liu et al., 2010).
- Therapeutic window uncertainty. The margin between antitumor efficacy and neurotoxicity remains undefined for most phytochemicals. PINK1 inhibition, while potentially sensitizing tumors to therapy, may induce neuronal death given PINK1’s essential role in dopaminergic neuron survival (O’Flanagan and O’Neill, 2014; Petit et al., 2005). GPR55 antagonism could theoretically affect CNS immune surveillance or neuroinflammatory responses with unknown consequences.
- Formulation challenges. Achieving therapeutic CNS concentrations requires advanced delivery systems (nanoparticles, liposomes, intranasal administration, convection-enhanced delivery) that add regulatory complexity and manufacturing costs (Wang et al., 2022; Agnihotri et al., 2016).

### Clinical translation landscape

As of December 2024, no Phase III trial has demonstrated improved overall survival with phytochemical monotherapy in GBM (Weller et al., 2017). The clinical evidence landscape comprises:

- Early-phase cannabinoid trials.
  ○ NCT03529448 (CBD + TMZ), NCT05753007 (CBD for glioma-related seizures), and NCT05629702 (cannabis-based medicine) are ongoing or recently completed, with results pending. These trials primarily assess safety and tolerability, with efficacy endpoints underpowered for definitive conclusions.
  ○ TTX101 (piperlongumine hydrogel). This FDA orphan drug-designated product represents the most advanced plant-derived therapy specifically targeting GBM, designed for local post-surgical application. Phase I/II trials (NCT number pending publication) will establish local tolerability and preliminary efficacy, but systemic administration of piperlongumine faces the pharmacokinetic barriers detailed above.
- Methodological limitations. Published cannabinoid trials in glioma are predominantly single-arm, non-randomized, or retrospective, with heterogeneous patient populations, concomitant therapies, and outcome measures. The absence of biomarker-stratified trials (e.g., selecting patients based on GPR55 expression, PINK1 status, or metabolic profile) limits mechanistic interpretation and reproducibility.

### Integration of authors’ preliminary data

Preliminary *in silico* and *in vitro data* from our group (Turizo Smith et al., 2025) suggesting cannabinoid modulation of PINK1 and GPR55 must be interpreted within these limitations. Computational docking studies predict binding interactions but cannot confirm functional consequences; cell-based assays demonstrate viability effects but do not establish mechanism specificity, BBB penetration, or *in vivo* relevance (Turizo Smith et al., 2025). These findings are presented as hypothesis-generating rather than therapeutically validated, requiring independent replication in orthotopic models with pharmacokinetic confirmation before clinical relevance can be asserted (Delgado-López et al., 2020; Wang et al., 2022).

### Recommendations for future research

Addressing these limitations requires establishing standardized preclinical evaluation using orthotopic PDX or GEMM models with pharmacokinetic-pharmacodynamic correlation; brain penetration assessment using *in vitro* BBB models (hCMEC/D3 cells) and *in vivo* microdialysis or PET imaging; formulation development for enhanced CNS delivery (lipid nanoparticles, exosome encapsulation, focused ultrasound BBB disruption); biomarker-driven clinical trial design stratifying patients by GPR55/PINK1 expression, metabolic phenotype, or tumor molecular subtype and regulatory science investment to establish quality control standards for complex botanical preparations or achieve synthetic accessibility of lead compounds.

## Conclusion

**FIGURE 4:**
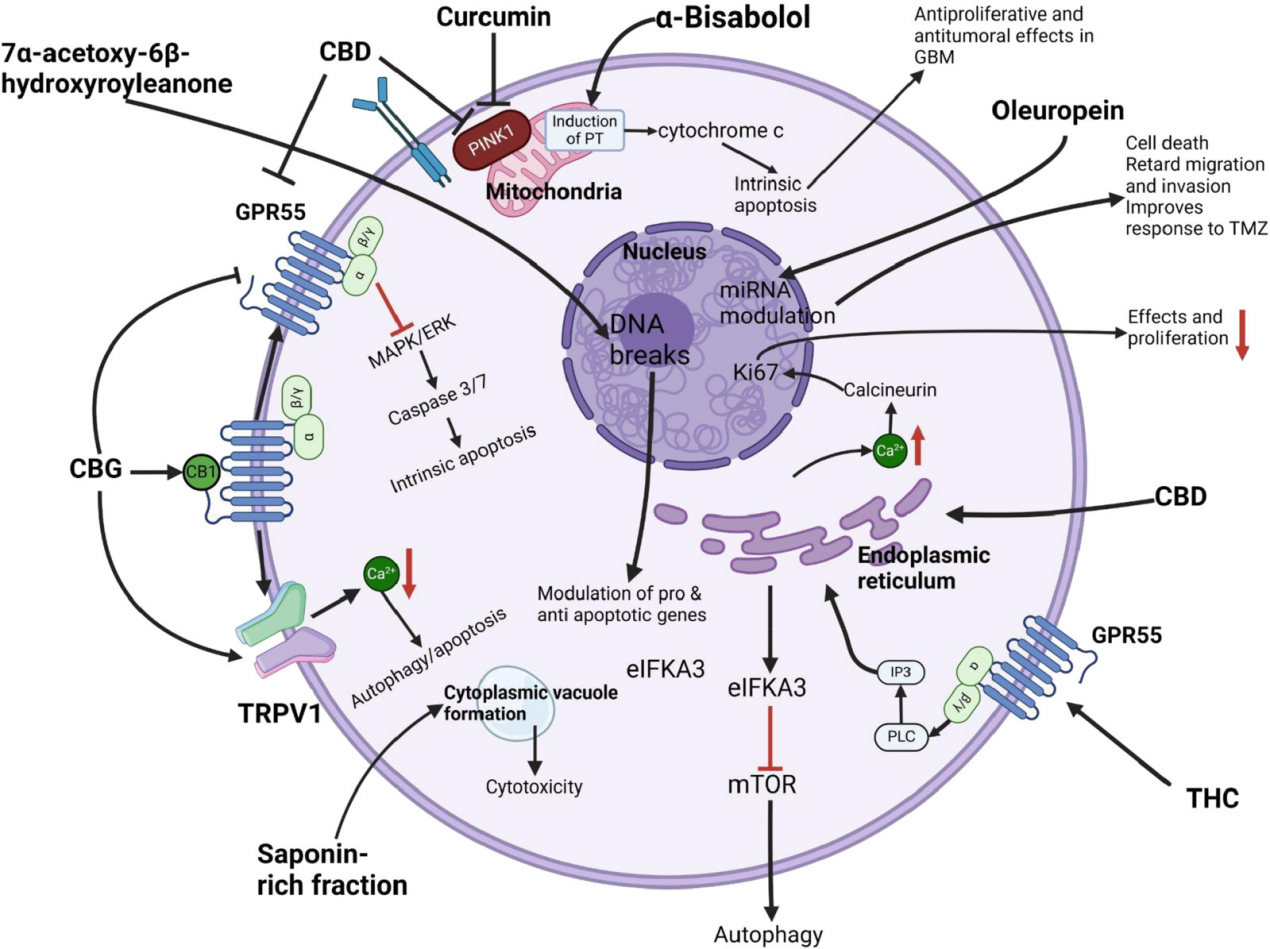
This figure illustrates the proposed mechanisms of action for various bioactive compounds on cellular processes related to apoptosis, autophagy, and cytotoxicity. CBG interacts with CB1 (Cannabinoid receptor type 1) and GPR55 (G protein-coupled receptor 55), modulating the MAPK/ERK (Mitogen-Activated Protein Kinase/Extracellular Signal-Regulated Kinase) pathway and activating caspases 3 and 7 to induce intrinsic apoptosis. Additionally, CBG activates TRPV1 (Transient Receptor Potential Vanilloid 1), influencing calcium ion (Ca^2+^) homeostasis to regulate autophagy and apoptosis. The diterpene 7α-acetoxy-6β-hydroxyroyleanone acts on mitochondria by inducing permeability transition (PT), leading to cytochrome c release and intrinsic apoptosis. α-Bisabolol enhances mitochondrial-mediated apoptosis, while oleuropein promotes cell death, retards migration and invasion, and improves the response to temozolomide (TMZ), a chemotherapeutic agent. These compounds also influence DNA damage and miRNA modulation, affecting proliferation markers such as Ki67. CBD acts on the endoplasmic reticulum, increasing intracellular calcium levels and activating calcineurin, which modulates pro- and anti-apoptotic gene expression. CBD also inhibits mTOR (Mechanistic Target of Rapamycin) via eIFKA3 (Eukaryotic Translation Initiation Factor Kinase 3), thereby promoting autophagy. THC activates the PLC (Phospholipase C) pathway through IP3 (Inositol 1,4,5-Trisphosphate), influencing calcium release from the endoplasmic reticulum. The saponin-rich fraction induces cytoplasmic vacuole formation, contributing to cytotoxicity. This summary highlights the multifaceted actions of these compounds on key cellular pathways, supporting their potential therapeutic applications in cancer and related diseases. Finally, CBD and curcumin could inhibit PINK1 (PTEN Induced Kinase 1) and the PI3K/Akt/mTOR pathway, leading to inhibition of mitophagy, along with the regulation of related proteins such as PINK1, which may trigger antitumor and antiproliferative effects. Biorender was used to construct the figure.

GBM is the most common malignant brain tumor in adults, which continues to be incurable with low median survival rates. Most clinical trials with chemotherapeutic agents have failed, partially due to their poor penetration through the BBB (Vengoji et al., 2018). In this regard, the beneficial effects of plant extracts or synthetic drugs derived from these can modify such limitation, thereby altering BBB permeability (Ferber et al., 2011), and may act as potential modulators of other therapeutic targets, including GPR55 and PINK1, which can improve prognosis in GBM cases (Figure 4).

As summarized in the present review, there are several preclinical data to support further studies on the use of plant derivatives in gliomas, and due to the current characteristics of this type of cancer, it demands that we explore alternative therapies to improve patient outcomes and survival. Prospective randomized clinical trials should be conducted to explore the use of plant extract therapy to improve health outcomes derived from these conditions, and on the other hand, to evaluate their potential use as adjunctive agents in standard treatments.

Notably, recent research such as TTX101, NCT05753007, NCT05629702, and NCT03529448 underscores the importance of plant-based research in glioblastoma treatment. TTX101, which has received orphan drug designation by the FDA, is a hydrogel containing piperlongumine, an alkaloid from Piper longum, designed for application post-surgically to target residual GBM cells due to its pro-apoptotic properties. NCT03529448, NCT05753007, and NCT05629702 are clinical trials investigating cannabinoid-based therapies, including CBD and THC, for their potential to inhibit glioma cell survival, modulate immune response, and reduce inflammation. These studies highlight the promise of plant-derived therapies to address GBM’s treatment challenges, offering innovative approaches to improve drug delivery, target specific pathways, and potentially enhance patient outcomes and survival.
